# An analytical study on changes in university students' attitudes toward physical activity

**DOI:** 10.3389/fspor.2024.1323582

**Published:** 2024-09-06

**Authors:** JingJing Nie

**Affiliations:** ^1^ State Key Laboratory of Cognitive Neuroscience and Learning, Beijing Normal University, Beijing, China; ^2^ State Key Laboratory of Cognitive Neuroscience and Learning, Faculty of Psychology, Beijing Normal University, Beijing, China

**Keywords:** attitudes toward physical activity change, implicit attitude, explicit attitude, physical activity, university students

## Abstract

**Purpose:**

Positive attitudes toward physical activity have been consistently linked to health Behaviors. This study aims to validate the effectiveness of attitude change in the domain of exercise and identify the mechanisms through which this change occurs in the context of sports and promote exercise behavior.

**Methods:**

103 university students (20.7 ± 1.97 years, 40 males, and 63 females) completed the implicit and explicit attitude tests. Based on their exercise levels assessed using the Physical Activity Rating Scale (PARS-3), participants were categorized into low and high exercise level groups. From this sample, 66 university students (19 males and 47 females) with low exercise levels completed a self-generated information task. Subsequently, physical activity was assessed through a skipping rope task.

**Results:**

The implementation of the self-generated information task resulted in notable alterations in implicit attitudes (*η*i^2^ = 0.07) toward physical activity during subsequent Implicit Attitude Test (IAT) evaluations. Additionally, significant changes were observed in explicit attitudes (*η*e^2^ = 0.12) toward physical activity among university students. Moreover, the use of self-generated information positively influenced immediate exercise behavior.

**Discussion:**

This study provides valuable insights into the impact of attitudes on behaviors among university students. It suggests that attitudes toward physical activity can be effectively changed through the use of self-generated information methodology, leading to an enhancement in immediate exercise behavior. Such attitude change has the potential to promote healthier behaviors and reduce the risk of diseases. These findings hold implications for interventions aimed at enhancing physical activity levels among university students.

## 1 Introduction

It is possible that someone may ask you, “Do you think I look good today?” Your response may be influenced by your personal attitudes towards exercise and health, as well as a preference for an active and fit lifestyle. Sometimes, we may not be fully aware of the attitudes we hold, yet they significantly impact our behavior and how we perceive the world around us. Attitude change is a fundamental concept in the study of individual behavior (1). Health behavior is not solely determined by the conscious processing of information but is also influenced by automatic processes (2). This perspective offers fresh insights into the factors that promote health-protective behaviors, such as engaging in physical activity (3). Theories on health behavior focus on the role of attitudes as antecedents to behavior, particularly the influence of both explicit and implicit attitudes on predicting and changing behavior. Implicit and explicit cognitive processes are incorporated within dual-process models (4). The Associative-Propositional Evaluation model [APE (5)] describes implicit and explicit cognitive processes, providing a framework to understand the potential connections between automatic evaluations and exercise behavior.

### 1.1 Associative propositional evaluation (APE) model

Gawronski and Bodenhausen (5) proposed that implicit and explicit attitudes are two distinct types of cognitive processing and representation. The APE (Associative-Propositional Evaluation) model views the assessment of implicit attitudes as a process of associative processing. Associative processing is influenced by external stimuli and the activation of associations in memory. For instance, a chance encounter with an individual who is considered unattractive at a charity event may activate a positive impression of that person. Conversely, encountering the same individual in a dark alleyway may activate feelings of hostility towards him. The same stimulus can elicit different patterns of association in memory, leading to diverse emotional responses based on the influence of external stimuli and the structure of associations in memory. In a study conducted by Jabold (6), the author examined stereotyping against criminals and law-abiding individuals, two groups that clearly differ in valence.

In contrast, explicit attitudes are assessed through propositional processing, which is influenced by the accuracy of the information activated by associations and guided by logical reasoning. Unlike associative processing, propositional processing distinguishes between correctness and error. In the associative processing system, automatic emotional responses are transformed into evaluative judgments regarding the liking or disliking of an object, forming corresponding propositions.

**Figure 1:**
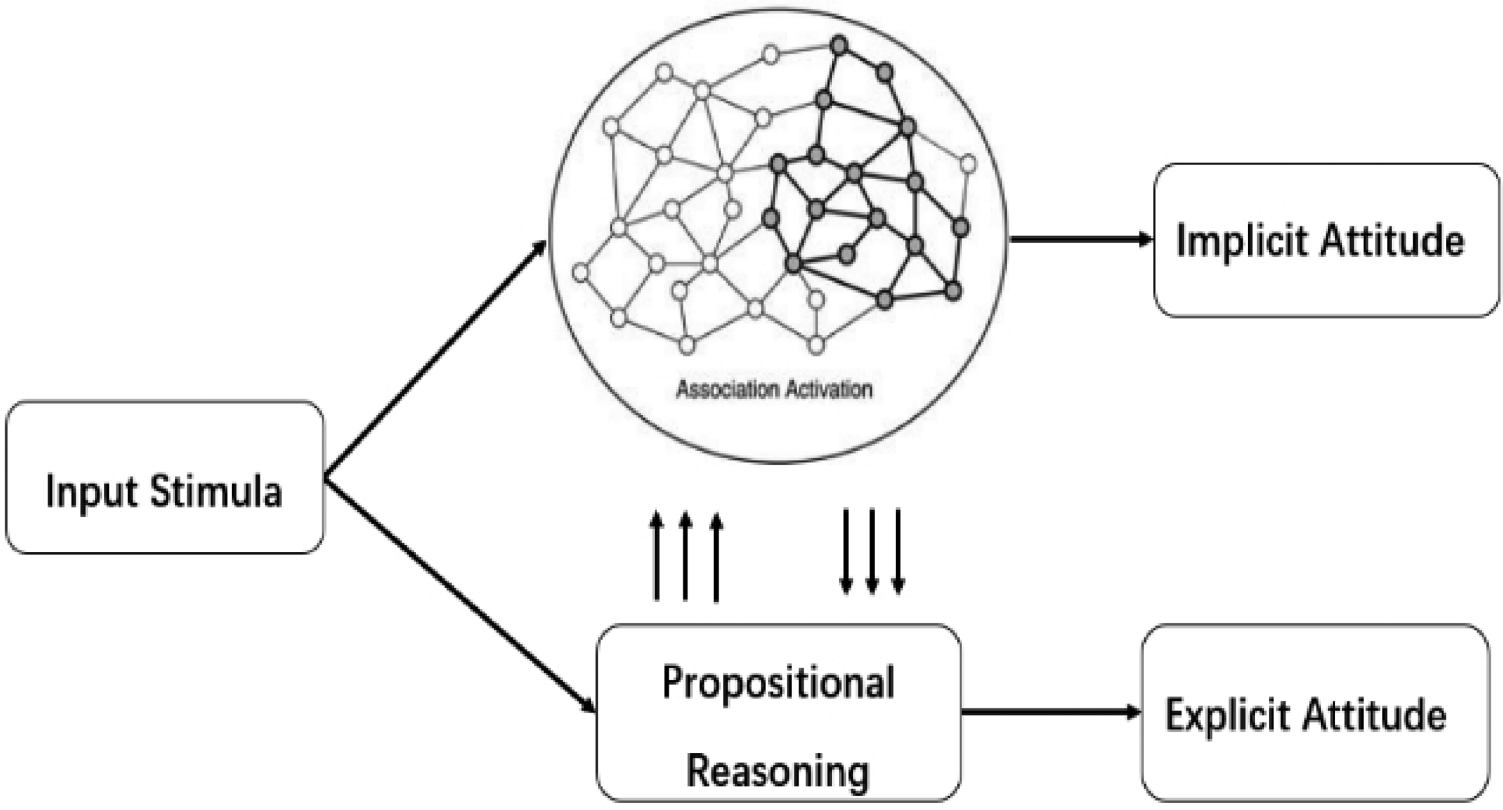
Mechanism of action of the associative proposition evaluation model (7).

The APE model, proposed by Gawronski and Bodenhausen (7) and illustrated in Figure 1, suggests that associative and propositional processing can occur independently or interactively under varying circumstances. The propositional perspective has already stimulated considerable research on automatic evaluation (8). The APE model is a research paradigm in attitude change that employs the self-generated information task (9). This paradigm is widely utilized in social psychology research and has demonstrated effectiveness in moderating stereotypes and influencing both implicit and explicit attitudes under specific experimental conditions (5).

### 1.2 The present research

The role of associative propositional evaluation (APE) models in attitude change has garnered support from several studies. For instance, Chang and Ko (10) examined the impact of celebrity endorsement on consumers’ implicit and explicit attitudes toward brand choice using the APE model. Their findings revealed that celebrity endorsement messages activate brand-related information, leading to implicit changes in consumers’ attitudes and ultimately influencing their choice of branded products.

Multiple studies have examined the influence of explicit and implicit attitudes on physical activity behavior (3, 11, 12, 35). Rebar et al. (11) demonstrated that physical activity is partly determined by non-conscious processes such as habits, automatic associations, and priming effects. Biondolillo and Pillemer (13) showed that implicit attitudes toward physical activity can be modified, and positive implicit attitudes can be elicited through pleasant exercise representations, although the effects may be short-term. Additionally, it has been found that activating autobiographical representational memory can enhance physical activity behavior, with representations often serving as motivational incentives for high-level athletes (14). Other studies have explored how representations can enhance physical activity behavior (15), and Dasgupta and Greenwald (16), among others, found that presenting individuals with sample pictures of a different nature could alter their automatically generated implicit cognitive biases. Consequently, a substantial body of research suggests that both implicit and explicit attitudes can be altered through specific techniques.

### 1.3 Summary of hypotheses and objective

Based on Greenwald and Banaji’s (17) implicit social cognition theory and Gawronski and Bodenhausen’s (5) APE model, this study aims to assess university students’ attitudes toward physical activity and to change their attitudes towards physical activity. First, a questionnaire survey and the Implicit Association Test will be used to analyze the differences in explicit and implicit attitudes towards physical activity among male and female university students with different levels of physical activity. Then, from the university students who participated in the initial tests, those with low levels of physical activity are selected. Using the self-generated information task paradigm of the APE model, the study examines the impact of self-generated information on the implicit and explicit attitudes of university students towards physical activity. Therefore, this study aims to test the following hypotheses:

(H1) University students with high levels of exercise would exhibit more positive implicit and explicit attitudes toward physical activity compared to those with low levels of physical activity. (H2) Male university students would have more positive external and implicit attitudes toward physical activity than female university students. (H3) There would be a relationship between students’ explicit and implicit attitudes toward physical activity. (H4) When self-generating a favorite sports star, participants would have more positive implicit and explicit attitudes toward physical activity compared to when generating a disliked sports star. (H5) The effect of self-generated associative information on participants’ implicit and explicit attitudes toward physical activity would be influenced by the amount of information. (H6) Participants who self-generated information as like/dislike sports stars would exhibit a tendency to engage in more sports exercise behaviors.

By investigating these hypotheses, the study aimed to contribute to a more comprehensive understanding of the relationship between attitudes, self-generated information, and physical activity among university students. According to the APE model, mental representations of attitudinal goals encompass distinct associations that cannot be simultaneously activated. To enhance the positive affective experience of individuals with low exercise levels and consequently influence their attitude towards physical activity, the activation of exercise-related knowledge associations becomes crucial. In this regard, self-generated associative information, such as generating preferences for liked or disliked sports stars, serves to activate preexisting positive or negative exercise-related knowledge associations stored in memory. This activation leads to the formation of new positive associations that directly impact implicit attitudes, subsequently influencing explicit attitudes. The present study aims to investigate the effects of self-generated like/dislike sports star information on university students’ implicit and explicit attitudes towards physical activity, with the intention of enhancing the implicit and explicit attitudes of individuals with low exercise levels. The ultimate objective is to predict and improve exercise levels.

## 2 Method

First, assessed the exercise levels among university students, and the attitudes towards physical activity selecting those with low physical activity levels. Subsequently, Based on the APE model, it explained changes in implicit and explicit attitudes towards physical activity. Investigated how self-generated associative information (liking/disliking sports stars) and the amount of self-generated information influenced the attitude activation of low-level exercisers. The study found that self-generated positive information could improve both implicit and explicit attitudes towards physical exercise among university students, and further explored the impact of university students’ exercise attitudes on their physical activity.

### 2.1 Participants

A total of 156 general university students from a university in Beijing were recruited for this study. Participants were required to complete the Physical Activity Rating Scale honestly. From the initial sample, 103 participants (male to female ratio of 4:6, age 20.7 ± 1.97 years). Among them, 30% had low levels of exercise, while 70% had high levels of exercise. Among the participants with low levels of exercise, based on the results of the Implicit Association Test, data from 4 participants were excluded due to careless responses and missing answers. This left 66 valid participants (male to female ratio of 3:7, age 20.5 ± 2.03 years) were involved in the self-generated information task. All participants reported no physical or vision-related diseases that could potentially impact their participation. Moreover, all participants voluntarily agreed to take part in the experiment, demonstrated familiarity with computer operations, and had not previously participated in similar experiments. This rigorous screening process aimed to ensure a homogeneous sample of university students with varying levels of exercise and without any confounding health conditions.

### 2.2 Measures

103 participants used the Physical Activity Rating Scale to measure their level of exercise, the IAT Program to measure implicit attitudes, and the TPB Attitude Scale to measure explicit attitudes towards physical activity. Physical Activity Rating Scale (PARS-3) revised by Liang (18), the exercise volume of the subjects participating in physical activity in one year was assessed from three aspects: intensity, time and frequency. Exercise volume = intensity × time × frequency. Exercise volume evaluation criteria: ≤19 points for small exercise volume; 20–42 points for moderate exercise volume; ≥43 points for large exercise volume, and two groups of high and low exercise levels are screened out. The reliability of the Physical Activity Rating Scale was 0.82.

This study employed the IAT, which has been widely used in previous research to investigate implicit attitudes (19–21, 34). This test paradigm involves linking physical activity-related conceptual vocabulary and non-physical activity-related conceptual vocabulary with positive and negative attribute words. The IAT test was conducted using a Dell 14-inch LCD color display with a resolution of 1,366 × 768. The experimental program was created using Inquisit 3.0.2 professional IAT software. The test consisted of seven stages, with the target words displayed in green and the attribute words in white. The font used was Arial, and the background was black. The category labels were positioned in the upper left and upper right corners of the screen, while the stimulus words were presented in the center. The participants were seated approximately 40 cm away from the screen. The IAT program followed an evaluation-type design, and the specific procedure is detailed in Table 1.

**Table 1:** Evaluative IAT.

Step	Task description	Number of trials	Key response	
			Left button	Right button
1	Practice initial concept word identification	20	Physical activity glossary	Non-physical Activity glossary
2	Practice associative attribute word identification	20	Positive	Negative
3	Practice linking task 1	20	Positive + physical activity	Negative + physical activity
4	Formal linkage task 2	40	Positive + physical activity	Negative + physical activity
5	Practice identifying opposite attribute words	20	Negative	Positive
6	Practice opposite linkage task 1	20	Negative + physical activity	Positive + physical activity
7	Formal opposite linkage task 2	40	Negative + physical activity	Positive + physical activity

The TPB Attitude Scale from The Planned Behavior Scale was used to measure the participants’ explicit attitudes. The TPB Attitude Scale consists of instrumental and affective attitude measures, including 2 instrumental attitude items (useful-useless, sensible-stupid), 3 affective attitude items (interesting-boring, relaxing-tense, happy-painful) on a seven-point scale. The reliability of the TPB Attitude Scale was 0.74. Assessments that take into account implicit and explicit attitudes may be affected by the order of measurement. After the subjects completed the implicit attitude test, the explicit attitudes were measured (22).

A self-generated information task was employed, using 2 (self-generated associative information: like, dislike sports stars) × 2 (quantity: 2, 7) between-subjects experimental design. The independent variables were the self-generated associative information and its quantity. The dependent variables were the participants’ scores on implicit and explicit attitudes towards physical exercise, as well as their behavioral intention scores. Participants (*n *= 66) with low levels of exercise, were randomly assigned to one of the four groups. (1) listing 2 names of sports stars they liked, (2) listing 7 names of sports stars they liked, (3) listing 2 names of sports stars they disliked, and (4) listing 7 names of sports stars they disliked. The distribution of participants in each group was as follows: 17 participants in the 2 names liked group, 16 participants in the 7 names liked group, 17 participants in the 2 names disliked group, and 16 participants in the 7 names disliked group. The specific implementation steps are as follows:

Step 1: Self-generated information task. Participants were instructed to recall and list either 2 or 7 sports stars they liked or disliked. The following guiding phrases were provided:

“Please carefully recall 2 sports stars you like. Please list the names of these 2 sports stars in the space below.”

“Please carefully recall 7 sports stars you like. Please list the names of these 7 sports stars in the space below.”

“Please carefully recall 2 sports stars you dislike. Please list the names of these 2 sports stars in the space below.”

“Please carefully recall 7 sports stars you dislike. Please list the names of these 7 sports stars in the space below.”

Step 2: Measurement of implicit attitudes using the Implicit Association Test (IAT).

Step 3: Explicit attitude measure.

Step 4: Measurement of exercise behavior. Participants were presented with a choice between engaging in a cognitive task or skipping rope. They were then given one minute of free skipping.

### 2.3 Data analysis

SPSS 22.0 was used for statistics and analysis of the results. Descriptive statistics were calculated for parametric variables, including mean values and standard deviations (SD).Paired sample t test and two-way analysis of variance were used. The relationship between the two factors was analyzed using correlation analysis. Data were analyzed using non-parametric statistical tests. Statistical significance was accepted as *p* < 0.05.

## 3 Results

### 3.1 Implicit and explicit attitudes towards physical activity

A paired sample *t*-test was conducted to compare the performance on the compatible and incompatible tasks in the evaluative IAT test for implicit attitudes towards physical activity. The relevant statistical results are presented in Table 2. The results presented in Table 2 indicate a highly significant implicit effect of the evaluative IAT test (*t* = −18.02, *df* = 102, *p *< 0.01). These findings demonstrate that university students tend to associate physical exercise with positive attributes and hold a positive attitude towards physical exercise. Moreover, the implicit attitudes towards physical exercise among university students with high exercise levels are more positive compared to those with low exercise levels. This pattern of results provides clear support for Hypothesis 1. To further explore the relationship between exercise level, gender, and the implicit test of the evaluative IAT, a two-way ANOVA was conducted. Exercise level and gender were used as independent variables, while the evaluative IAT served as the dependent variable. The means for each condition are presented in Table 3.

**Table 2:** Comparison of differences between evaluated IAT compatible and incompatible task response times.

	Different tasks	*M*	*SD*	*t*	*p*
	Compatible	846.74	203.48		
Evaluated IAT T				−18.02	0.00
	Incompatible	1,276.98	336.22		

**Table 3:** Descriptive statistics of high and low exercise levels and gender-specific university students in evaluative IAT scores.

Exercise level	Gender	*M*	*SD*	*n*
High	Male	551.96	309.52	21
	Female	657.59	150.65	12
Low	Male	302.58	132.58	19
	Female	374.16	205.11	51

The ANOVA results showed (see Table 4) that the main effect of exercise level was significant, *F* (1, 99) = 30.16, *p *< 0.05, *η*^2 ^= 0.23; the main effect of gender was marginally significant, *F* (1, 99) = 3.34, *p *> 0.05, *η*^2 ^= 0.03; the interaction between exercise level and gender was not significant, *F* (1, 99) = 0.12, *p *> 0.05, *η*^2 ^= 0.02. The results show that university students with high exercise level had higher implicit attitudes in evaluative IAT than those with low exercise levels, and that university students of both genders had significant margins in the Evaluative IAT.

**Table 4:** Analysis of variance (ANOVA) for high and low exercise level and gender-specific university students in evaluative IAT scores.

Source of variation	*df*	*F*	*p*	*η* ^2^
Exercise level	1	30.16	0.00	0.23
Gender	1	3.34	0.07	0.03
Exercise level × gender	1	0.12	0.73	0.02

A two-way ANOVA was conducted with high and low exercise levels and different genders of university students as independent variables and explicit attitudes as dependent variables. Means are displayed in Table 5. The results of the ANOVA (Table 6) showed that the main effect of exercise level was significant, *F* (1, 99) = 21.82, *p *< 0.01, *η*^2^ = 0.18; the main effect of gender was significant, *F* (1, 99) = 4.53, *p* < 0.05, *η*^2^ = 0.04; the interaction of exercise level and gender was not significant, *F* (1, 99) = 0.47, *p* > 0.05, *η*^2^ = 0.01. The results indicate that there is a significant difference between high and low exercise level university students in terms of their externalised attitudes, with higher exercise level university students (*M*_male _= 6.62, *M*_female _= 5.91) having higher externalised attitudes than low exercise level university students (*M*_male _= 5.28, *M*_famale _= 4.92). This pattern of results clearly supported H1. There was a significant difference between the gender of the university students in terms of their explicit attitudes, with male university students having more positive exogenous attitudes than females. This pattern of results clearly supported H2.

**Table 5:** Descriptive statistics on explicit attitude scores of high and low exercise levels and male and female university students.

Exercise level	Gender	*M*	*SD*	*n*
High	Male	6.62	0.55	21
	Female	5.91	1.01	12
Low	Male	5.28	0.79	19
	Female	4.92	1.37	51

**Table 6:** Analysis of variance (ANOVA) for high and low exercise level and gender-specific university students’ explicit attitude scores.

Source of variation	*df*	*F*	*p*	*η^2^*
Exercise level	1	21.82	0.00	0.18
Gender	1	4.53	0.03	0.04
Exercise level × gender	1	0.47	0.49	0.01

A correlation analysis of the implicit and explicit attitudes towards physical activity among university students (see Table 7) showed that there was a significant positive correlation between the explicit attitudes and the evaluative IAT (*r *= 0.25). This indicates that implicit and explicit attitudes are significantly correlated. Hofmann et al. (36) meta-analysis of 126 studies with 53 different contents showed a correlation of 0.24 between implicit association measures and explicit measures. Nosek (37) analysed a large sample of data obtained from a web-based survey and found that the correlation coefficient between implicit and explicit attitudes fluctuated in the range of 0.01–0.62. This also demonstrates that implicit attitudes are not completely unconscious in the APE model and that there is a relationship between implicit and explicit attitudes. The results of the correlation clearly supported H3.

**Table 7:** Correlation analysis between indicators of implicit and explicit tests.

	Evaluated IAT	External attitude
Evaluated IAT	1	
External attitude	0.25	1

### 3.2 Self-generated associative information

**Figure 2:**
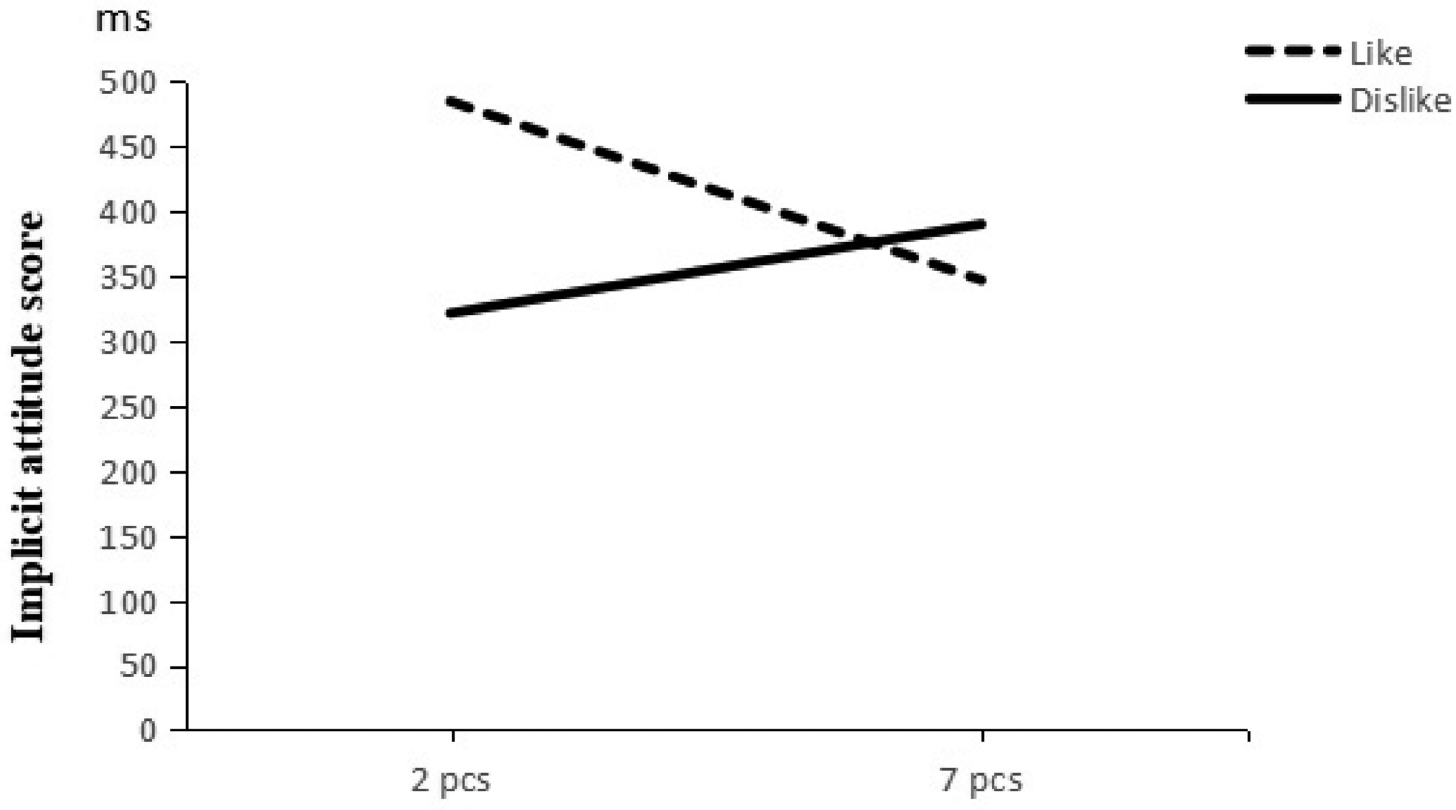
Interaction between self-generated associative information and quantity on implicit attitudes.

A two-way ANOVA was conducted using the generation of associative information and quantity as independent variables and the score of implicit attitudes as dependent variables (Descriptive results are shown in Table 8). The results of the ANOVA revealed (see Table 9) that the main effect of subjects’ self-generated associative information was significant *F* (1, 62) = 4.41, *p* < 0.05, *η*^2^ = 0.07, and that subjects’ implicit attitudes towards physical activity were significantly higher when they self-generated information about liking sports stars than in the self-generated disliked sports stars condition. The results of the correlation clearly supported H4. The main effect of the quantity of self-generated associative messages by subjects was not significant *F* (1, 62) = 0.30, *p* > 0.05, *η*^2^ = 0.01, and the interaction between generated associative messages and quantity of implicit attitudes was significant *F* (1, 62) = 6.08, *p* < 0.05, *η*^2^ = 0.09 (see Figure 2).

**Table 8:** Descriptive statistics of self-generated associative information and quantity of implicit attitude scores.

Associated Information	Quantity	*M*	*SD*	*n*
Like	2 pcs	484.44	157.87	17
	7 pcs	346.78	159.84	17
Dislike	2 pcs	321.26	124.95	16
	7 pcs	389.90	132.75	16

**Table 9:** Analysis of variance for self-generated associative information and quantity of implicit attitude scores.

Source of variation	*df*	*F*	*p*	*η^2^*
Associated information	1	4.41	0.04	0.07
Quantity	1	0.30	0.59	0.01
Associated Information × quantity	1	6.08	0.01	0.09

A simple effects test showed that there was a significant difference between liked/disliked sports stars on implicit attitudes towards physical activity when the self-generated associative information was 2*, F* (1, 62) = 10.4, *p* < 0.05; the effect on implicit attitudes towards physical activity when liked/disliked sports stars did not reach a significance level when the self-generated associative information was 7, *F*(1, 62) = 0.07, *p* > 0.05. This indicates that the generation of 2 like sports stars had a positive effect on the implicit attitude towards physical activity among university students. The results of the correlation clearly supported H5.

**Figure 3:**
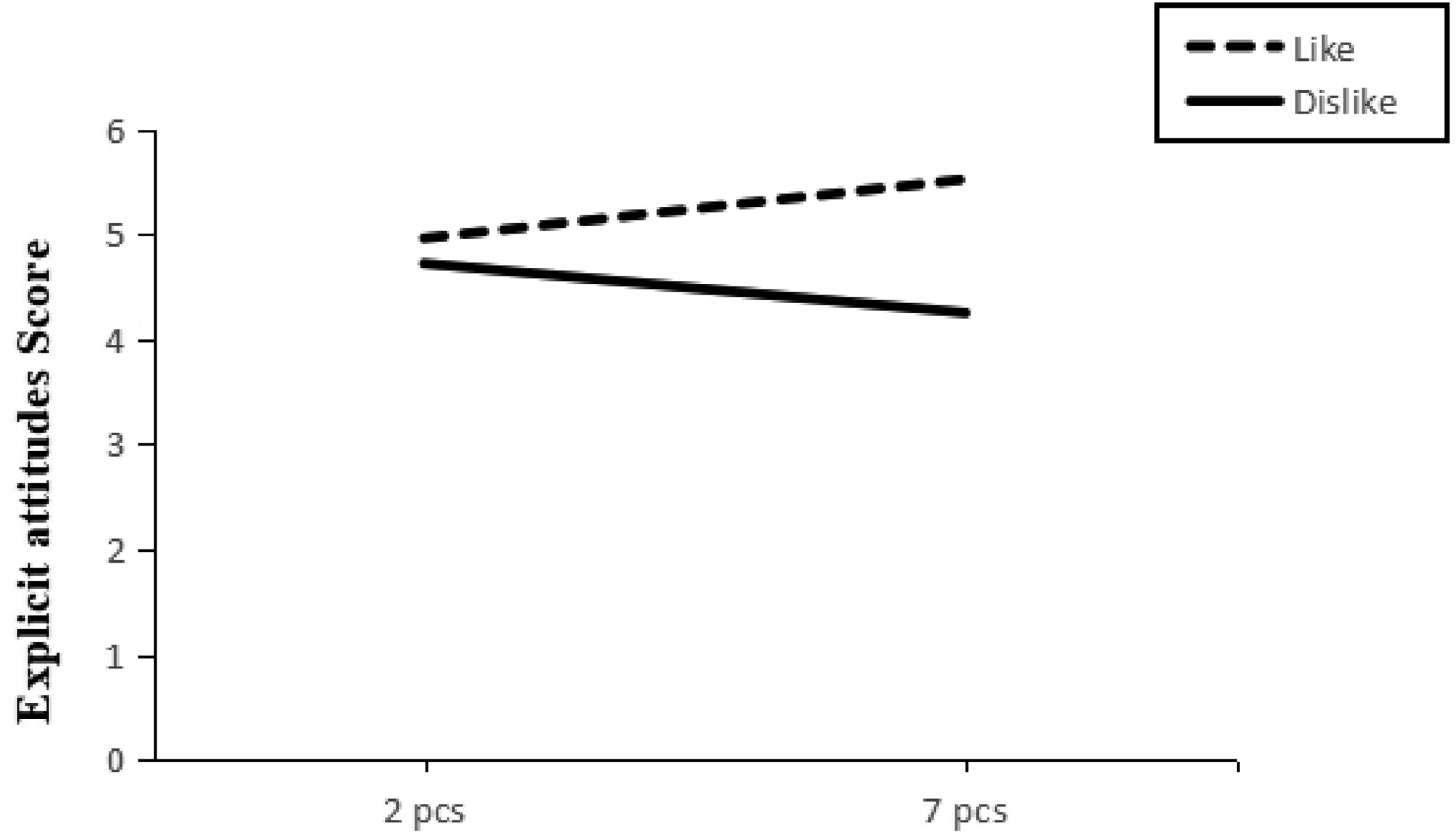
Interaction between self-generated associative information and quantity on explicit attitudes.

A two-way ANOVA was conducted with self-generated associative information and quantity as the independent variables and scores of explicit attitudes towards physical activity as the dependent variables (Descriptive results are shown in Table 10). The results of the ANOVA showed (see Table 11) that the main effect of the subject generating associative information was significant *F* (1, 62) = 9.37, *p* < 0.01, *η*^2^ = 0.12, and that the subject's explicit attitude towards physical activity was significantly higher in the self-generated like sports stars than in the self-generated dislike sports stars condition (*M*_Like2_ = 4.96, *M*_Dislike2_ = 4.72; *M*_Like7_ = 5.52, *M*_Dislike2_ = 4.25), see Table 12, clearly supported H4. The main effect of the quantity of associative messages generated by the subjects was not significant *F* (1, 62) = 0.30, *p* > 0.05, *η*^2^ = 0.00, and the interaction between the quantity of associative messages generated and the number of explicit attitudes was significant *F* (1, 62) = 6.08, *p *< 0.05, *η*^2^ = 0.06 (see Figure 3).

**Table 10:** Descriptive statistics of self-generated associative information and quantity of explicit attitude scores.

Associated information	Quantity	*M*	*SD*	*n*
Like	2 pcs	4.96	0.96	17
	7 pcs	5.52	1.01	17
Dislike	2 pcs	4.72	1.25	16
	7 pcs	4.25	0.92	16

**Table 11:** ANOVA of self-generated asociation information and quantity in explicit attitude scores.

Source of variation	*df*	*F*	*p*	*η* ^2^
Associated information	1	9.37	0.00	0.12
Quantity	1	0.03	0.86	0.00
Associated information × quantity	1	4.00	0.05	0.06

**Table 12:** Chi-square analysis of self-generated information and behavioral choices.

Self-generated information	Test skipping rope		Total
Like sports stars	10 (13.8)	21 (17.2)	31
Dislike sports stars	18 (14.2)	14 (17.8)	32
Total	28	35	63

A simple effects test showed that subjects had a significant difference between liked/disliked sports stars in their explicit attitudes toward physical activity when generating seven associative messages *F* (1, 62) = 12.2, *p *< 0.05; liked/disliked sports stars did not reach a significance level in their explicit attitudes towards physical activity when generating two associative messages *F* (1, 62) = 0.43, *p* > 0.05. This indicates that the self-generation of 7 like sports stars had a positive effect on the explicit attitude towards physical activity. The results of the correlation clearly supported H5.

### 3.3 Physical activity

After initiating the attitudes towards physical activity of self-generating preferences for like/dislike sports stars, the participants were given two options: to choose to continue with the test or engage in one minute of free skipping. Using self-generated information (like/dislike sports stars), the participants’ behavioral choices (skipping rope/test) were subjected to a chi-square test (see Table 12). The results of the chi-square test indicated *χ*^2^ = 3.67, *p *< 0.05. This suggests that self-generated sports stars information is associated with the behavior of skipping rope, and participants are more likely to choose skipping rope as their preferred activity. The results of the correlation clearly supported H6.

## 4 Discussion

The current study aimed to promote physical activity among university students by changing their attitudes towards physical activity through self-generated information. In measuring university students’ attitudes towards physical activity, the research findings indicate a significant impact of university students’ implicit attitudes towards physical exercise as measured by the IAT. Specifically, there is a stronger association between physical exercise vocabulary and positive attributes, leading to faster responses. Moreover, university students with high exercise levels demonstrate more positive implicit and explicit attitudes towards physical exercise compared to those with low exercise levels. These results align with previous studies, such as the review conducted by Rebar et al. (11), which highlighted the correlational nature of the evidence linking physical activity and implicit attitudes.Additionally, male participants exhibit more positive explicit attitudes towards physical exercise compared to females. These differences may stem from traditional concepts and social stereotypes, with males placing greater emphasis on physical attributes like body shape, strength, and muscles. Such factors contribute to variations in attitudes towards physical exercise between genders. Importantly, there is a significant correlation between university students’ IAT scores and their explicit attitudes (*r *= 0.25). This finding aligns with Hofmann, Gawronski, and Schmitt’s (2005) meta-analysis, which reviewed 126 research reports covering a wide range of topics. The meta-analysis indicated that the correlation between implicit association measurement and explicit attitudes fluctuates between 0.01 and 0.62. This result provides a fresh perspective for investigating the relationship between implicit and explicit attitudes and supports the notion that implicit attitudes in the APE model are not entirely unconscious, but rather interconnected with explicit attitudes. The formation of an exercise habit is a process that transitions from conscious effort to unconscious routine. At the beginning of an physical activity, conscious effort and attention are required. After maintaining it for a period of time, it can eventually become a habit, at which point the need for conscious effort and attention will decrease (23).

Implicit attitudes were previously believed to be stable and socially ingrained evaluative representations (24, 25). However, research has shown that situational factors can influence implicit attitudes (5). Conroy and Berry (26) consider automated assessments of exercise and physical activity as “potentially modifiable targets” for specific interventions. The findings of the current study demonstrate that self-generated associative information related to physical activity, such as liked or disliked sports stars, acts as a situational factor that influences both implicit and explicit attitudes towards physical activity.

In this study, self-generated associative information about sports stars effectively enhanced positive or negative associations in the participants’ memory systems regarding implicit attitudes towards physical activity, resulting in changes in implicit attitudes in subsequent IAT tests among university students. Recent arguments propose that associative activation can reconstruct implicit attitudes (27). The activation of self-generated liked/disliked sports star information induced specific positive/negative association patterns with physical activity, influencing both implicit and explicit attitudes towards physical activity. This suggests that contextual cues elicit distinct patterns of associative activation and that self-generated associative information can influence implicit attitudes (28, 29). Consequently, implicit attitudes can be modified through self-generated associative information.

Furthermore, researchers have examined the impact of celebrity endorsements on attitudes and product choices. Moran et al. (30) found that relational information has a stronger impact on non-automatic stereotypes compared to automatic stereotypes. Craeynest et al. (31) found that changes in implicit attitudes toward food and exercise had some small impact on obesity treatment in adolescents. A meta-analysis provides evidence that implicit attitudes toward physical activity are positively associated with physical activity in adults to a small degree (12). Based on the APE research on attitudes, Janiszewski Kuo and Tavassoli (32) suggest that attention should be focused on capturing consumer attention and enhancing consumers’ cognitive association with the brand (athlete and product). Studies have also indicated that implicit and explicit attitudes can predict health-related behaviors (33). The current study supports the research hypothesis that self-generated information can enhance immediate exercise behavior.

## 5 Limitations

In this study, the influence of attitude on immediate exercise behavior was found to be significant. However, further investigation is needed to explore the lasting effects of attitude on exercise behavior, with the goal of fostering long-term engagement in physical exercise and forming a healthy exercise habit. the study only provided an initiation of attitudes, and the sustained effect of this initiation needs to be verified. Future studies could expand the selection of participants for the study by choosing different regions, different universities, or different age groups as experimental participants.

## 6 Conclusion

Attitudes are malleable, and the APE model finds support in the domain of physical activity, where associative processing can influence both implicit and explicit attitudes towards physical activity. The study reveals that university students hold positive implicit and explicit attitudes towards physical activity, with those having a high exercise level exhibiting more positive implicit and explicit attitudes compared to those with a low exercise level. Moreover, male university students demonstrate more positive explicit attitudes towards physical activity than their female counterparts. The introduction of self-generated information regarding their favorite sports stars further enhances university students’ implicit and explicit attitudes towards physical activity. Notably, changes in attitude exhibit a certain predictive effect on exercise behavior and can temporarily foster increased engagement in physical activity, which helps promote university students’ active participation in physical activity.

## Data Availability

The raw data supporting the conclusions of this article will be made available by the authors, without undue reservation.
